# Wnt4 is significantly upregulated during the early phases of cisplatin-induced acute kidney injury

**DOI:** 10.1038/s41598-018-28595-4

**Published:** 2018-07-12

**Authors:** Yi-Xin He, Tian-Tian Diao, Shu-Min Song, Cen-Cen Wang, Yu Wang, Chun-Lan Zhou, Yi-Bing Bai, Shan-Shen Yu, Xuan Mi, Xin-Yu Yang, Qiu-Ju Wei, Bing Li

**Affiliations:** Department of Nephrology, the Second Affiliated Hospital of Harbin Medical University, Harbin, People’s Republic of China

## Abstract

Wnt4 is a secreted growth factor associated with renal tubulogenesis. Our previous studies identified that renal and urinary Wnt4 are upregulated following ischemia-reperfusion injury in mice, but the roles of Wnt4 in other forms of acute kidney injury (AKI) remain unclear. Here, we investigated the changes in Wnt4 expression using a cisplatin-induced AKI model. We found that renal and urinary Wnt4 expression increased as early as 12 hours, peaked at day 4 following cisplatin-induced AKI and was closely correlated with histopathological alterations. By contrast, the serum creatinine level was significantly elevated until day 3, indicating that Wnt4 is more sensitive to early tubular injury than serum creatinine. In addition, renal Wnt4 was co-stained with aquaporin-1 and thiazide-sensitive NaCl cotransporter, suggesting that Wnt4 can detect both proximal and distal tubular injuries. These data were further confirmed in a clinical study. Increased urinary Wnt4 expression was detected earlier than serum creatinine and eGFR in patients with contrast-induced AKI after vascular intervention. This study is the first to demonstrate that increased expression of renal and urinary Wnt4 can be detected earlier than serum creatinine after drug-induced AKI. In particular, urinary Wnt4 can potentially serve as a noninvasive biomarker for monitoring patients with tubular injury.

## Introduction

Acute kidney injury (AKI) has become a worldwide health problem due to its high morbidity, mortality and cost. According to a recent epidemiological study, AKI occurs in approximately 13.3 million people per year and contributes to approximately 1.7 million deaths because of delayed diagnosis and therapy^1^. In 2013, the International Society of Nephrology put forward the global AKI target of “0by25” to help improve the diagnosis and treatment of AKI globally^2^. A Chinese cross-sectional survey showed that AKI has a very high non-recognition rate (74.2%) and delayed AKI recognition is an independent risk factor for in-hospital mortality^3^. Currently, the diagnosis of AKI mainly depends on serum creatinine, which is an insensitive and nonspecific indicator of renal injury, because clear increases in this traditional AKI marker is only observed during the advanced stage of renal damage^4,5^. Therefore, using serum creatinine may delay early diagnosis and effective AKI treatment, resulting in either renal replacement therapy or death. The identification of more reliable, earlier biomarkers of tubule injury is urgently needed to facilitate early intervention and decrease AKI mortality.

The Wnt proteins belong to a highly conserved family of secreted growth factors that contain approximately 19 members in mammals^6,7^. The Wnt pathway is a complex cell-to-cell communication pathway involved in many embryonic and fetal developmental processes, including cell fate specification, differentiation, and phenotype regulation^8,9^. In our previous study, we found that the Wnt proteins are promptly and dramatically upregulated after ischemic kidney injury^10^. Among the Wnt family members, Wnt4 induces the mesenchymal-to-epithelial transition and is associated with tubulogenesis^11,12^. Mice lacking Wnt4 activity fail to form pretubular cell aggregates and completely lack tubular development^13^. Many studies have demonstrated that kidney repair is rapidly activated after kidney injury^10,14^. Our previous data showed that damage and repair simultaneously exist in tubular injury after ischemia-reperfusion injury (IRI)^15^. In our very recent study, we found that the expression of renal Wnt4 and urinary Wnt4 increased as early as 3 hours and peaked 24 hours after ischemia/reperfusion injury, whereas the serum creatinine level began to increase at 6 hours^15^. These results suggest that Wnt4 might be a more sensitive biomarker than serum creatinine for the early detection of tubular injury. Nephrotoxic agents, including chemotherapy drugs, contrast agents, antibiotics, biological agents and other drugs, are among the most common causes of AKI^16,17^. While the induction of Wnt4 in IRI models is well established, the response of Wnt4 to other forms of acute tubular injury has not been identified.

In this study, we investigated Wnt4 expression in a rat model of cisplatin-induced AKI and patients with contrast-induced AKI (CI-AKI) after vascular intervention. We demonstrated that the renal and urinary expression of Wnt4 markedly increased during the early stage of tubular injury. These results indicated that Wnt4 might be a more sensitive, noninvasive biomarker for detecting drug nephrotoxicity than serum creatinine. We suggest that Wnt4 may serve as a new biomarker for monitoring early tubular injury and repair processes.

## Results

### Kidney function and histological changes in a cisplatin-induced AKI model

**Figure 1:**
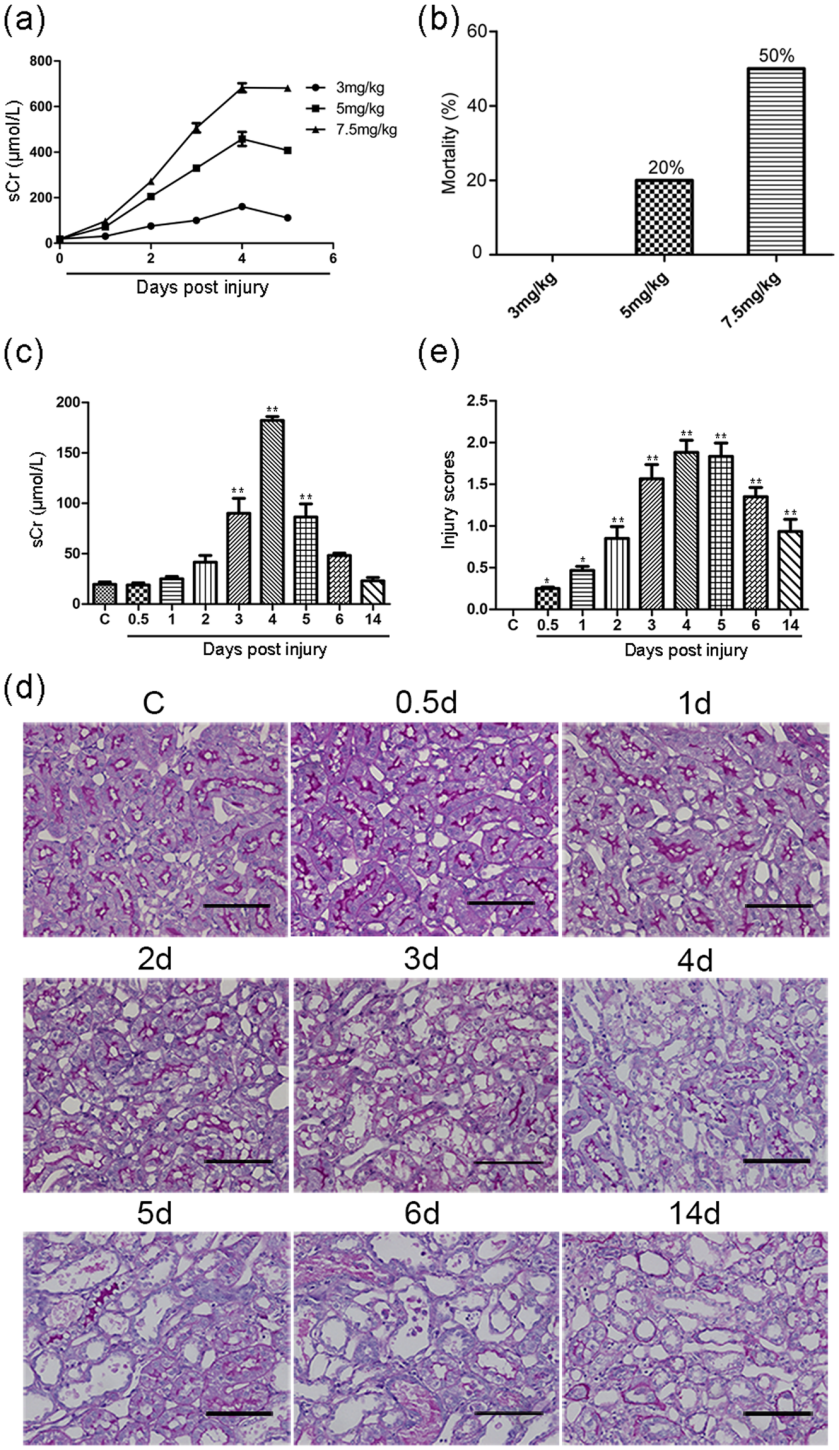
Kidney function and histological changes in the cisplatin-induced AKI model. (**a**) Serum creatinine level in different cisplatin dose groups in the pilot experiment. (**b**) Mortality in different cisplatin dose groups in the pilot experiment. (**c**) Serum creatinine level at different time points in the control and cisplatin-induced AKI rats (3 mg/kg). (**d**) Kidney histology as shown by periodic acid–Schiff staining (magnification, 200×). Bar = 100 μm. (**e**) Injury scores of kidney damage. Data are expressed as the mean ± SD. *p < 0.05, **p < 0.01 versus the control group (n = 6 per group). sCr, serum creatinine; C, control.

In our pilot experiments, we intraperitoneally injected rats with either 5 mg or 7.5 mg cisplatin/kg in 0.9% saline as previously described^18^. The serum creatinine level began to mildly increase at day 2 and peaked at days 4 or 5 in each dose group. The peak serum creatinine level in the 5 mg and 7.5 mg cisplatin/kg groups was up to 20- and 35-fold higher, respectively, than that at baseline (day 0) (Fig. 1a). However, mortality rates of 20% (5 mg/kg) and 50% (7.5 mg/kg) were observed during the first 6 days after the cisplatin administration (Fig. 1b), and severe tubular injury was observed in both cortex and medullary areas of the rat kidneys by periodic acid-Schiff staining (data not shown). To investigate earlier biomarkers of AKI, we decreased the dose of cisplatin to 3 mg/kg and successfully obtained an ideal model of drug-induced AKI with a suitable serum creatinine level and a zero-mortality rate. After the injection of 3 mg cisplatin/kg, the serum creatinine level in the rats significantly increased at day 3 and peaked at day 4 (~9-fold higher than that at baseline, Fig. 1c); then, the creatinine level restored at day 6. However, the histological examination revealed pathological changes, including swelling and vacuolization of the tubular epithelial cells, as early as 12 hours after the drug administration. Upon further progression, the renal tubular epithelium showed tubular lysis, dilation, disruption, necrosis, cast formation and cell sloughing in the lumen (Fig. 1d). The kidney tubular injury was scored using a semiquantitative method (Fig. 1e). The tubular injury peaked at day 4, and obvious injury was still observed at day 14 after the injection of cisplatin. At day 14, the focal atrophic tubules remained, and tubules with regeneration were also observed. In contrast, the serum creatinine level was not markedly increased until day 3 and rapidly restored to the baseline at day 6 after the cisplatin-induced AKI. The serum creatinine level could not precisely reflect the tubular injury. These data further demonstrate that serum creatinine has a low sensitivity in detecting kidney tubular injury. Therefore, identifying a sensitive biomarker for the detection of kidney tubular injury is highly desirable.

### Renal Wnt4 expression is more sensitive than serum creatinine to cisplatin-induced AKI and is closely correlated with tubular injury and Kim-1 expression

**Figure 2:**
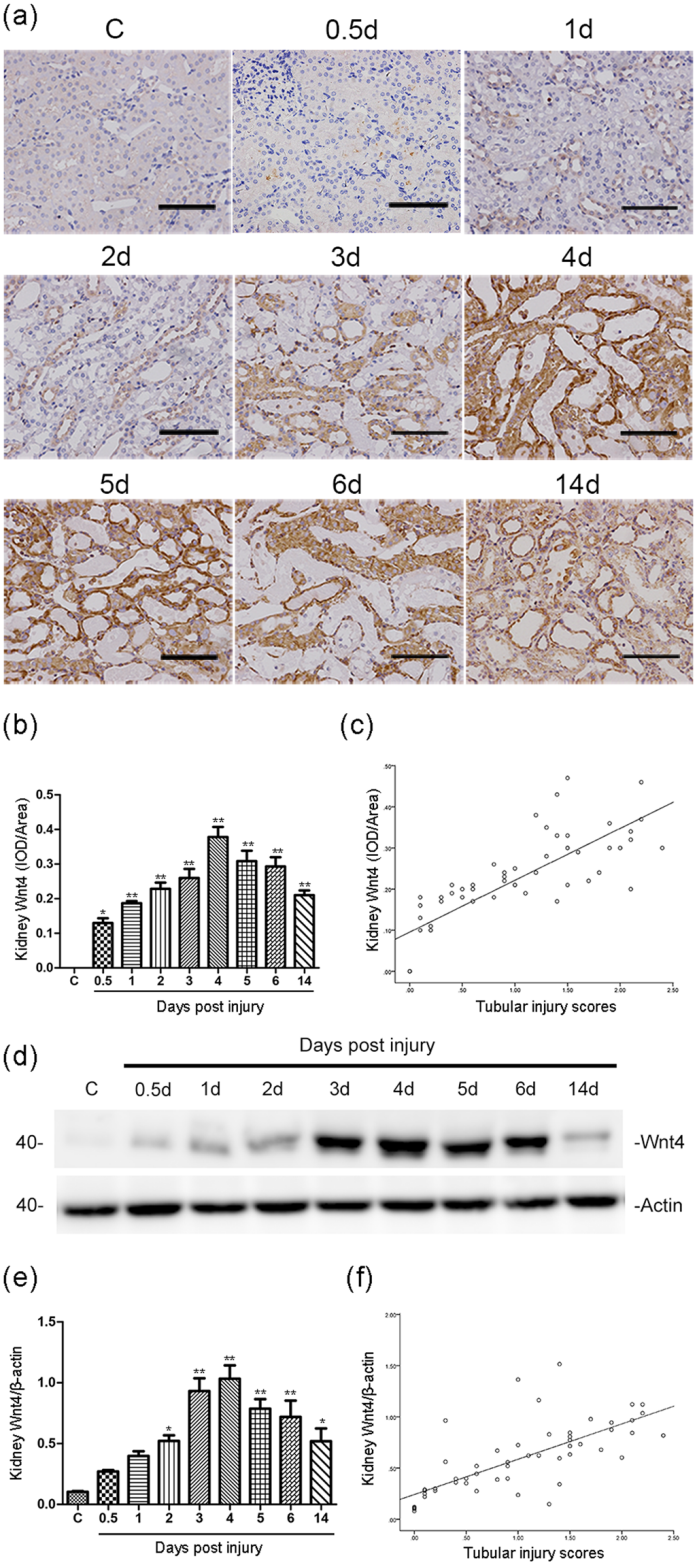
Kidney Wnt4 expression is significantly upregulated during the early stage of cisplatin-induced AKI and is correlated with tubular injury. (**a**) Representative immunohistochemical images showing kidney Wnt4 expression (brown) in the control and cisplatin-induced AKI rats at different time points (magnification, 200×). Bar = 100 μm. (**b**) Quantification of kidney Wnt4 expression in each group. Data are presented as the IOD/Wnt4-positive areas, as analyzed using Image-Pro Plus software. (**c**) Correlation between tubular injury and kidney Wnt4 expression by immunohistochemical staining (r^2^ = 0.795, *p* < 0.01). (**d**) Western blot assay of kidney Wnt4 expression in different groups. (**e**) Semiquantitative analysis showing the expression of Wnt4 in the kidney lysates after cisplatin injection. (**f**) Correlation between tubular injury and kidney Wnt4 expression by Western Blot (r^2^ = 0.72, *p* < 0.01). *p < 0.05, **p < 0.01 versus the control group (n = 6).

Our previous studies demonstrated that both the Wnt4 gene and protein are activated in the kidney after IRI and that the expression of renal Wnt4 increases much earlier than serum creatinine^15,19^. For detecting the role of Wnt4 in drug-induced tubular injury, we investigated the changes of renal Wnt4 expression in a cisplatin-induced AKI model. To confirm our hypothesis, we first performed immunohistochemical studies on kidney paraffin sections. As shown in Fig. 2a and Supplemental Fig. S1, no obvious Wnt4 expression was observed in the outer stripe of the outer medulla (OSOM) in the control group and healthy adult rats, but Wnt4 expression was significantly enhanced in the injured tubules as early as 12 hours after the cisplatin administration and reached a peak at day 4. Furthermore, Wnt4 expression remained significantly upregulated at day 14 compared with that in the control group. The same result is expressed graphically in Fig. 2b. In contrast, significantly increased levels of serum creatinine were not observed until day 3. Figure 2c shows the strong correlation between renal Wnt4 expression by immunostaining and kidney tubular injury. We further confirmed this finding by Western blot. As shown in Fig. 2d,e, obviously enhanced Wnt4 expression was observed by Western blot at 12 hours, and this expression was further increased at days 3 and 4 after the cisplatin injection. The Western blot results were consistent with the immunohistochemical staining results and showed that Wnt4 expression was highly correlated with kidney tubular injury (Fig. 2f).

Kim-1, a well-known biomarker of early acute renal tubular injury, showed a trend similar to that of Wnt4 in the cisplatin-induced AKI model. As shown in Supplemental Fig. S2a,b, kidney Kim-1 expression began to increase at 12 hours and peaked at day 4 after cisplatin administration. In addition, kidney Kim-1 expression was positively correlated with tubular injury and Wnt4 expression (Supplemental Fig. S2c,d). These data indicated that renal Wnt4 expression, like Kim-1, might be a more sensitive biomarker than serum creatinine in accurately reflecting the pathological changes that occur throughout the entire post-acute tubular injury process.

### Urinary Wnt4 is detected during the early stage of cisplatin-induced AKI and is correlated with renal Wnt4 expression and tubular injury

**Figure 3:**
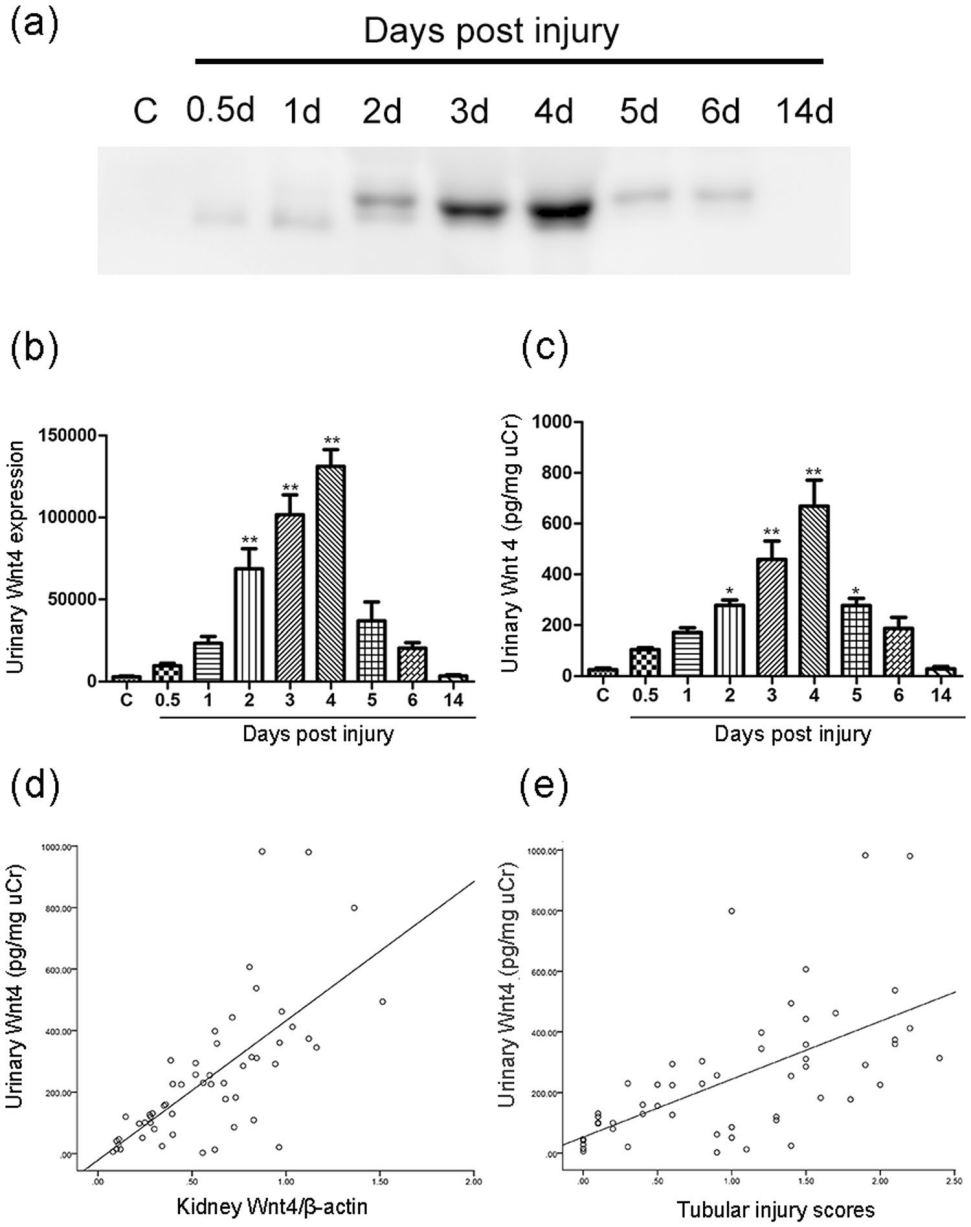
Urinary Wnt4 is detected during the early stage of cisplatin-induced AKI and is correlated with renal Wnt4 expression and tubular injury. (**a**) Western blot assay of urinary Wnt4 excretion in the control and cisplatin-induced AKI rats at different time points. (**b**) Data are presented as the intensity of urinary Wnt4 band analyzed using ImageJ software. (**c**) ELISA analysis of urinary Wnt4 normalized to uCr in each group. (**d**) Correlation between urinary Wnt4 and kidney Wnt4 expression (r^2^ = 0.699, *p* < 0.01). (**e**) Correlation between urinary Wnt4 and tubular injury (r^2^ = 0.615, *p* < 0.01). *p < 0.05, **p < 0.01 versus the control group (n = 6). uCr, urinary creatinine.

Wnt4 is a secreted glycoprotein required for nephrogenesis^20,21^. In previous studies, we detected increased urinary Wnt4 expression in both IRI and salt-sensitive hypertension (SSHT) models^15,22^, but the specific mechanism by which renal Wnt4 is secreted into the urine is still unknown. In the present study, using western blot and ELISA, we examined the changes in urinary Wnt4 expression in a cisplatin-induced AKI model. As shown in Fig. 3a, no obvious band was observed in the control group. However, a clear band appeared at 12 hours (much earlier than the serum creatinine level elevated), and the intensity of the Wnt4 expression gradually increased until day 4. Then, urinary Wnt4 declined during the repair stage and was undetectable at day 14. The same result is expressed graphically in Fig. 3b. To quantify the level of urinary Wnt4, we performed an ELISA and normalized the values to urinary creatinine. Similar results were confirmed by ELISA (Fig. 3c). In addition, we evaluated urinary Kim-1 expression by ELISA. Like Wnt4, Kim-1 expression elevated at 12 hours and peaked at day 4 (Supplemental Fig. S2e). The excretion of urinary Wnt4 was closely associated with kidney Wnt4 expression and urinary Kim-1 expression (Fig. 3d and Supplemental Fig. S2f). Both the urinary Wnt4 and Kim-1 levels were strongly associated with the severity of kidney tubular injury (Fig. 3e and Supplemental Fig. S2g). Altogether, these results demonstrated that the changes in urinary Wnt4 were consistent with tubular damage and that Wnt4 has the potential to serve as a noninvasive biomarker for the early detection of cisplatin-induced AKI.

### Wnt4 is expressed in all segments of cisplatin-injured tubules and can colocalize with macrophage markers

**Figure 4:**
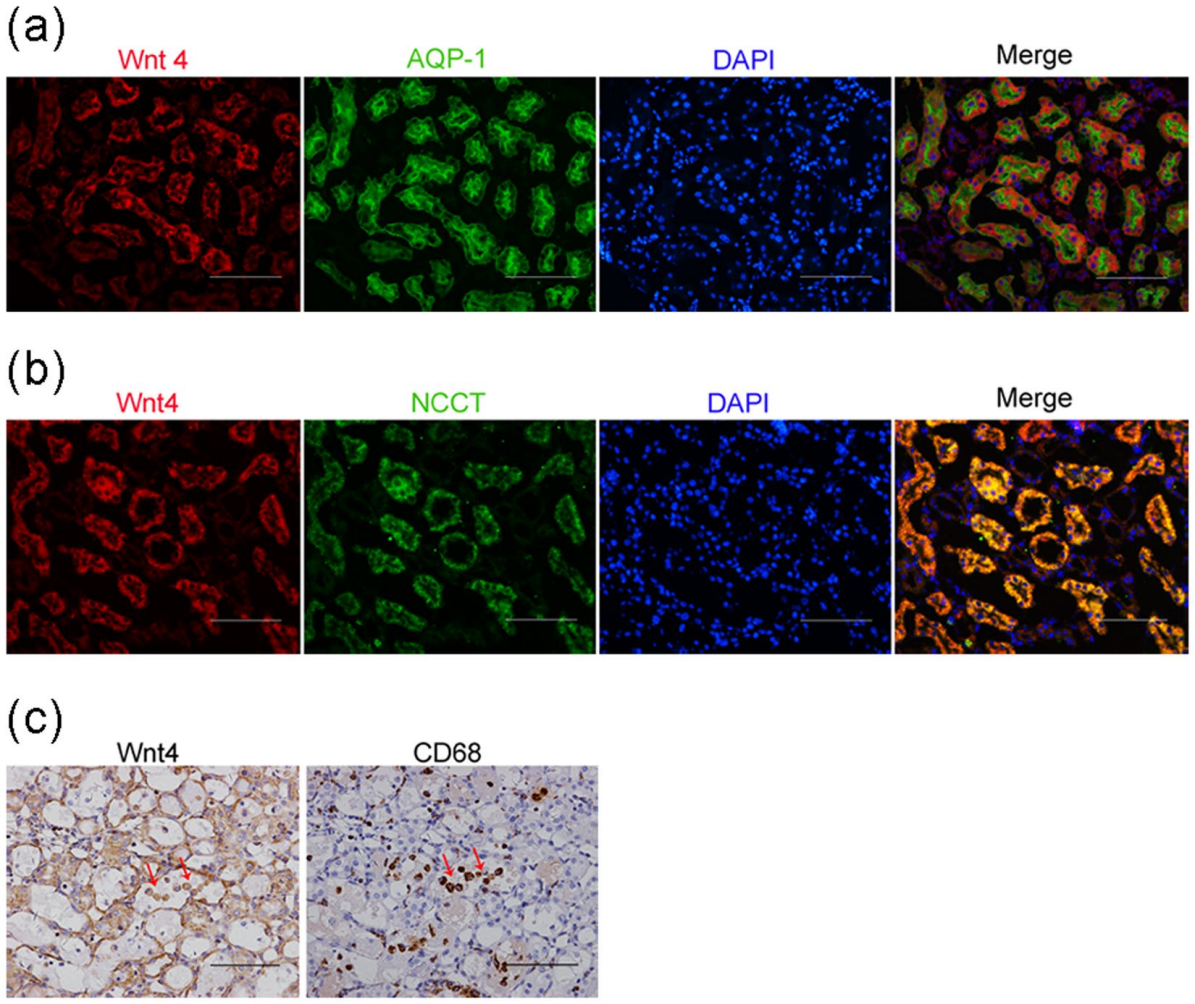
Wnt4 is expressed in both injured proximal and distal tubules after cisplatin treatment and colocalizes with CD68. (**a**) Co-staining of Wnt4 and the proximal tubular marker AQP-1. (**b**) Co-staining of Wnt4 and the distal tubular marker NCCT. (**c**) Wnt4 and CD68 staining in serial paraffin sections. Arrows showing cell debris expressing both Wnt4 and CD68. All magnification, 200×. All scale bars 100 μm.

To detect the expression pattern of Wnt4 in the cisplatin-induced AKI model, we co-stained Wnt4 with aquaporin-1 (AQP-1, a proximal tubular marker) and thiazide-sensitive NaCl cotransporter (NCCT, a distal tubular marker) and compared the localization with Kim-1. As shown in Fig. 4, Wnt4 co-localized with both AQP-1 (Fig. 4a) and NCCT (Fig. 4b), whereas Kim-1 only co-localized with injured proximal tubules as reported by previous study^23^ (Supplemental Fig. S3a,b). These results indicated that Wnt4 was activated in all injured tubules after the cisplatin injection. This behavior was in contrast to that of Kim-1, which was simply secreted by injured proximal tubules.

Macrophages are well known to play crucial roles in experimental and human renal disease, and these cells are implicated in the induction of injury, renal repair and fibrosis^24^. Our previous studies demonstrated that macrophages promote kidney repair via the Wnt signaling pathway and the upregulation of an autophagy protein that enhances the removal of necrotic debris^10,25^. Therefore, we investigated whether macrophages were involved in the secretion of Wnt4 by immunostaining. As shown in Fig. 4c, serial paraffin sections of the rat kidney after cisplatin treatment showed that certain cell debris in the dilated lumen expressed both Wnt4 and CD68. This colocalization indicated that certain macrophages may also secrete Wnt4 to participate in the repair after acute tubular injury. These data further confirmed our previous results in which macrophages promoted kidney repair via the Wnt signaling pathway.

### Both apoptosis and regeneration exist in injured tubular cells after cisplatin treatment, and both TUNEL-positive and Ki67-positive cells mainly co-stain with Wnt4

**Figure 5:**
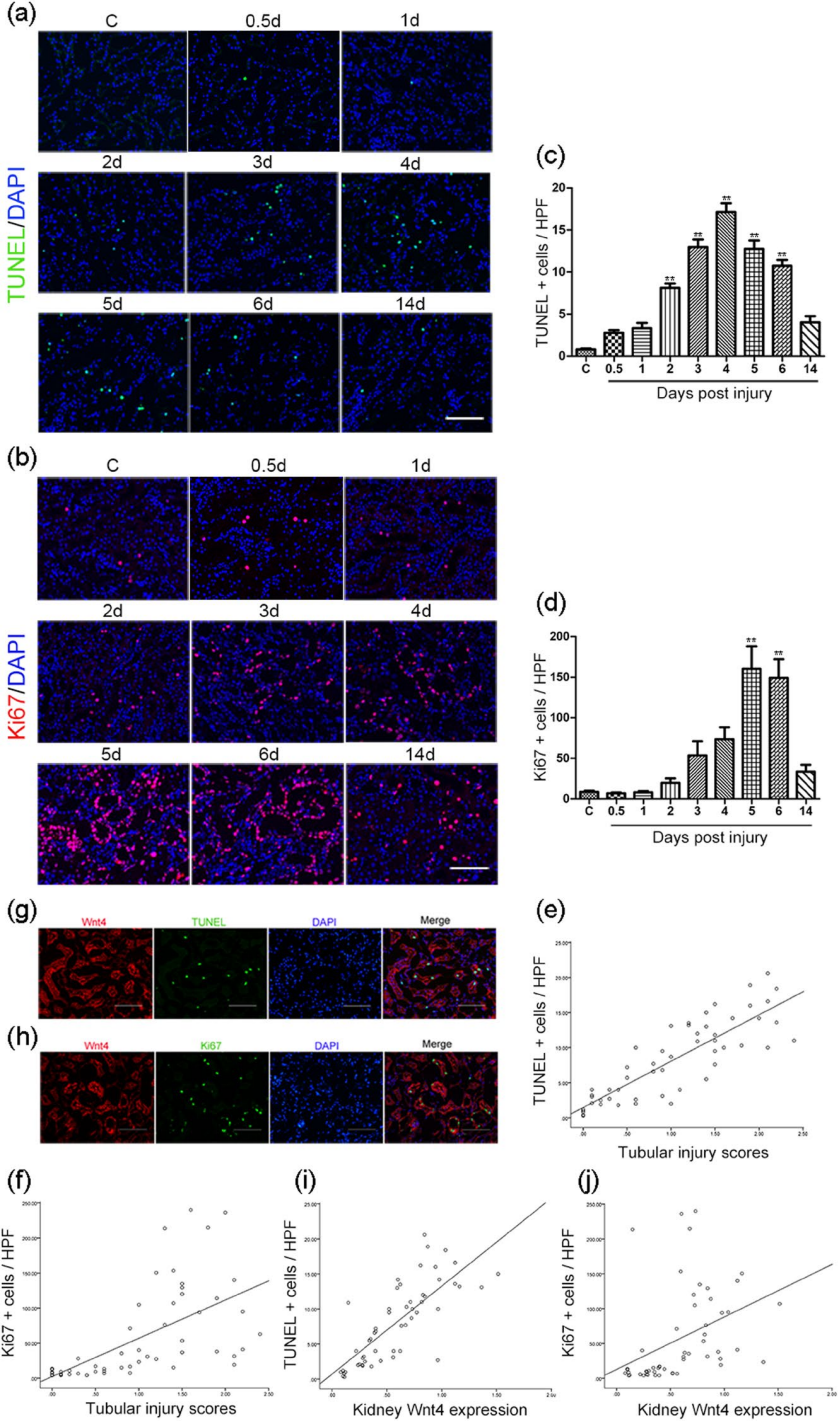
Both apoptosis and regeneration exist in injured tubular cells after cisplatin treatment, and both TUNEL-positive and Ki67-positive cells mainly co-stain with Wnt4. (**a**) Representative macrographs of TUNEL staining among the different groups (magnification, 200×). (**b**) Representative macrographs of Ki67 staining among the different groups (magnification, 200×). (**c**) Quantitative determination of TUNEL-positive cells in the OSOM region among the different groups. Data are presented as the number of TUNEL-positive cells per field (200×). (**d**) Quantitative determination of Ki67-positive cells in the OSOM region among the different groups. Data are presented as the number of Ki67-positive cells per field (200×). (**e**) Correlation between TUNEL-positive cells and tubular injury (r^2^ = 0.853, *p* < 0.01). (**f**) Correlation between Ki67-positive cells and tubular injury (r^2^ = 0.602, *p* < 0.01). (**g**) Co-staining of Wnt4 and TUNEL at day 4 after cisplatin administration (magnification, 200×). (**h**) Co-staining of Wnt4 and Ki67 at day 4 after cisplatin administration (magnification, 200×). (**i**) Correlation between kidney Wnt4 expression and TUNEL-positive cells after cisplatin administration (r^2^ = 0.777, *p* < 0.01). (**j**) Correlation between kidney Wnt4 expression and Ki67-positive cells after cisplatin administration (r^2^ = 0.397, *p* < 0.05). All scale bars 100 μm. **p < 0.01 versus the control group (n = 6).

Wnt4 is strongly associated with the mesenchymal-to-epithelial transition and tubulogenesis during the embryonic period. To detect the relationship between Wnt4 re-expression and epithelial cell apoptosis and the relationship between Wnt4 re-expression and tubule regeneration in the cisplatin-induced AKI model, we carried out the following immunofluorescence studies. First, we performed TUNEL and Ki67 (a cell proliferation marker) staining to investigate injury and regeneration in the tubular epithelium after cisplatin administration. As shown in Fig. 5a–d, there were few TUNEL-positive or Ki67-positive cells in the rat kidneys of the control group. In contrast, in the cisplatin group, the number of TUNEL-positive cells increased and peaked at day 4. In addition, the number of Ki67-positive cells in the OSOM region gradually increased and peaked 5 days after the cisplatin injection. A correlation analysis showed that both the TUNEL-positive and Ki67-positive cells were directly associated with tubular injury (Fig. 5e,f). These data suggested that damage and repair simultaneously exist in tubular epithelial cells after cisplatin exposure. Furthermore, as shown in Fig. 5g,h, the TUNEL-positive cells and Ki67-positive cells were mainly present in the tubular epithelium along with the upregulated expression of Wnt4. A correlation analysis indicated that both the TUNEL-positive and Ki67-positive tubular epithelial cells were associated with Wnt4 expression during the cisplatin-induced tubular damage period and the following recovery period (Fig. 5i,j). These results suggested that Wnt4 may undergo rapid activation after kidney injury and then involved in repair and regeneration.

### Increased urinary Wnt4 expression was detected earlier than alterations in serum markers in patients with CI-AKI

**Figure 6:**
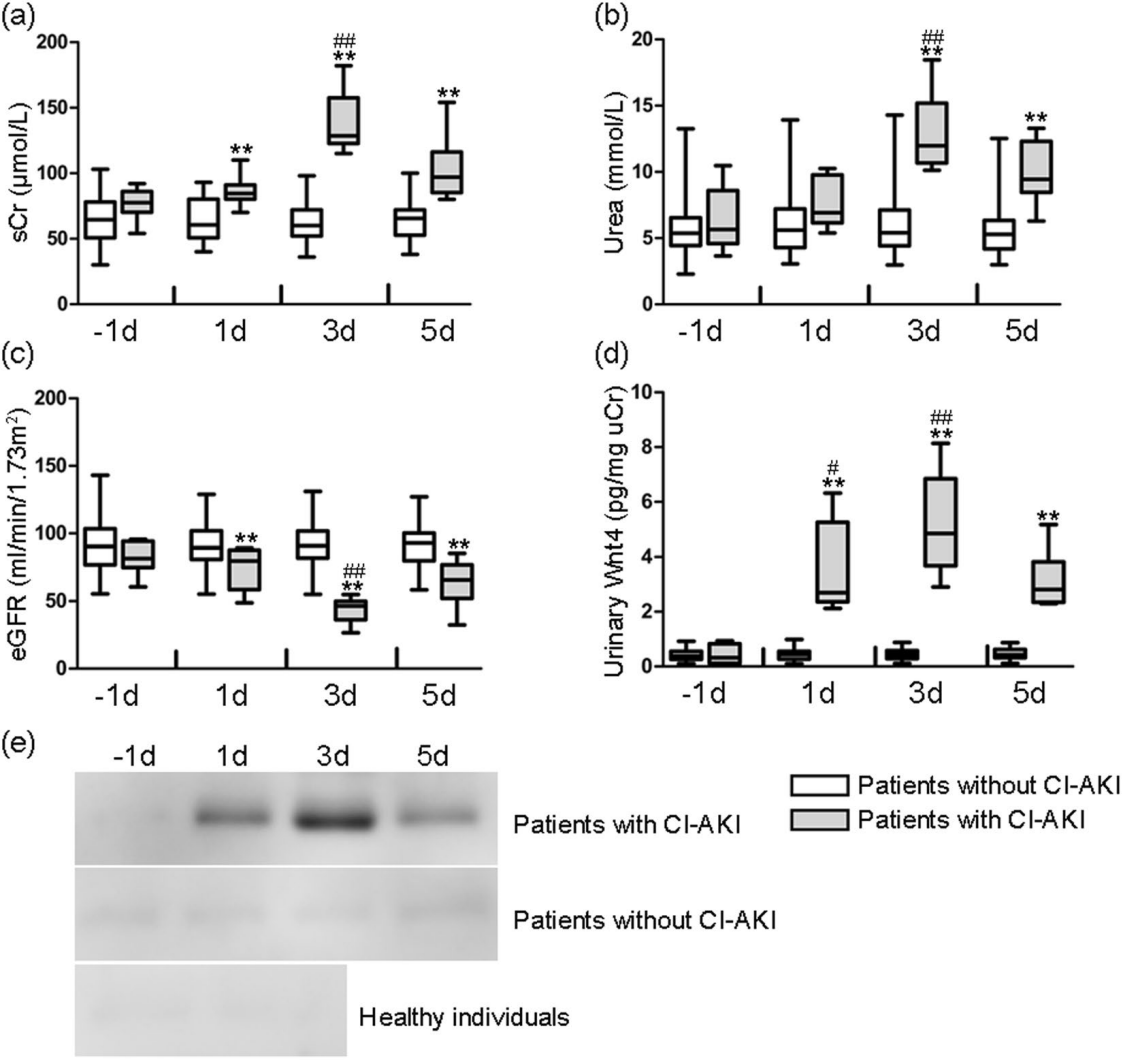
Urinary Wnt4 is elevated in patients with CI-AKI, earlier than alterations in serum creatinine, urea and eGFR. (**a**) Serum creatinine levels of patients with or without CI-AKI at different time points. (**b**) Urea levels of patients with or without CI-AKI at different time points. (**c**) eGFR of patients with or without CI-AKI at different time points. (**d**) ELISA analysis of urinary Wnt4 normalized to uCr in patients with or without CI-AKI at different time points. (**e**) Western blot assay of urinary Wnt4 excretion in patients with CI-AKI, patients without CI-AKI and healthy individuals. −1d, 1d, 3d and 5d refer to the time points of blood or urine sample collection of one day before and 1, 3 and 5 days after contrast agent administration. sCr, serum creatinine; uCr, urinary creatinine. **p < 0.01: patients with CI-AKI versus patients without CI-AKI at the same time point. ^#^*p* < 0.05, ^##^*p* < 0.01 versus the −1d group.

Contrast agents have the potential to cause short-term or even permanent renal injury, which is termed contrast-induced acute kidney injury (CI-AKI). To evaluate the clinical utility of urinary Wnt4 for detecting early AKI, we investigated urine samples of patients undergoing interventional treatments. Eighty-six patients with normal renal function and urinalysis results were enrolled in the study. Low-osmolar contrast agents were used in all patients. CI-AKI is usually defined as an increased serum creatinine level of at least 25% above baseline after the contrast administration^17^. According to the diagnostic criteria, 8 out of 86 suffered from CI-AKI (approximately 10%). The clinical characteristics of these patients are summarized in Table 1. The levels of serum creatinine and urea and eGFR in patients without CI-AKI were not obviously different before and after contrast agent administration. Significant alterations in serum creatinine, urea and eGFR in patients with CI-AKI were observed until day 3 after contrast agent administration and then decreased at day 5 (Fig. 6a–c). We performed western blot and ELISA to analyze patients’ urine samples obtained one day before and 1, 3 and 5 days after contrast agent administration. Both assays demonstrated that increased urinary Wnt4 expression was occurred as early as day 1 after contrast-induced AKI, and its level peaked at day 3 and then decreased but remained detectable at day 5 (Fig. 6d,e). However, we did not detect Wnt4 expression in the urine of healthy individuals or patients without CI-AKI (Fig. 6d,e). These results were consistent with the findings observed in our animal experiments and further confirmed that urinary Wnt4 has the potential to serve as a noninvasive biomarker for the early detection of drug-induced AKI.

**Table 1:** Basal clinical characteristics of patients undergoing interventional treatments.

Parameters	All	Patients with CI-AKI	Patients without CI-AKI	*P* value
Subjects	86	8	78	
Age (years)	67.5 (59–78)	70.5 (67.25–72.5)	67 (58.25–78)	0.721
Male	55 (64%)	6 (75%)	49 (63%)	0.767
Weight (kg)	65 (58–70.75)	64.5 (59–71)	65 (58–70.75)	0.988
SBP (mmHg)	126 (110.75–140)	134.5 (129.25–143.5)	122 (110–140)	0.127
DBP (mmHg)	78 (70–83)	75 (70–79.25)	78 (70–85.25)	0.677
Hemoglobin (g/L)	130 (116–147)	126.5 (105.5–135.75)	130 (119.25–147.75)	0.368
ALT (U/L)	26 (17–39.75)	19 (12.75–29.33)	26 (17.25–40)	0.11
AST (U/L)	31.5 (21–45.75)	29.5 (18.75–37)	32 (21.25–47.25)	0.618
Serum albumin (g/L)	40.05 (36–46.23)	34.3 (32.43–40.44)	40.95 (36.05–47.7)	0.015^*^
Urea (mmol/L)	5.38 (4.5–6.23)	5.66 (4.80–7.22)	5.38 (4.47–6.47)	0.645
Serum creatinine (μmol/L)	66 (54–78.75)	77.5 (70.75–84)	64.5 (51.25–78)	0.052
eGFR (mL/min/1.73 m^2^)	90.29 (75.81–100.47)	81.64 (75.14–91.75)	90.5 (77.94–102.9)	0.206
**Clinical diagnosis**				
Liver cancer	30 (34.9%)	2 (25%)	28 (35.9%)	0.821
Hepatic hemangioma	18 (20.9%)	2 (25%)	16 (20.5%)	0.766
Bronchiectasia	11 (12.8%)	2 (25%)	9 (11.5%)	0.716
Lung cancer	6 (7%)	1 (12.5%)	5 (6.4%)	0.52
Esophageal cancer	8 (9.3%)	1 (12.5%)	7 (9%)	0.744
Others	13 (15.1%)	0	13 (16.7%)	0.389
**Comorbidities**				
Diabetes mellitus	10 (11.6%)	0	10 (12.8%)	0.618
Hypertension	25 (29.1%)	2 (25%)	23 (29.5%)	0.79
Coronary heart disease	31 (36%)	3 (37.5%)	28 (35.9%)	0.928
Viral hepatitis B	24 (27.9%)	1 (12.5%)	23 (29.5%)	0.544
**Chronic medications**				
NSAIDs	6 (7%)	0	6 (7%)	0.932
ACEI/ARB	16 (18.6%)	4 (50%)	12 (15.4%)	0.055
**Contrast agents**				
Iohexol (35–122.5 g)	34 (39.5%)	4 (50%)	30 (38.5%)	0.798
Iopromide (100 mL)	52 (60.5%)	4 (50%)	48 (61.5%)	0.798

Data are given as the median and interquartile ranges. The dose of iohexol is different for treating different diseases. *P < 0.05 versus the group of patients without CI-AKI.

## Discussion

AKI is a disease that evolves from early injury to severe damage, leading to kidney failure and the need for renal replacement therapy. The window for early, targeted interventions in AKI is narrow. Insufficient and delayed recognition of AKI is considerably related to a rise in morbidity and mortality^3^. Serum creatinine, the current primary diagnostic method for AKI, is insensitive and can be influenced by various extrarenal factors, such as muscle mass, age, gender, and drug-induced interference^26,27^. Therefore, the prompt and effective management of AKI depends on more sensitive and specific biomarkers. The renal tubular epithelia undergo various forms of cell death after acute hypoxia or toxic injury^28–30^. This is followed by rapid, robust repair processes, which are characterized by the pronounced proliferation of tubular epithelial cells and the expression of growth factors^31^. Many renal injury models have identified the key genes and signaling pathways of kidney development that are re-expressed during regeneration, suggesting that there are similarities between development and repair^32–34^. Hence, the acute response to postnatal renal injury may represent a recapitulation of normal kidney development. Wnt signaling plays a key role in kidney morphogenesis, and previous studies have suggested that the expression of Wnt signaling is increased in renal epithelium in response to AKI^35^. During development, Wnt4 is required for completing the mesenchymal-to-epithelial transition. Therefore, it is conceivable that Wnt4 may re-express following a postnatal tubular injury. In the present study, we found that the expression of renal and urinary Wnt4 was considerably increased at 12 hours and markedly increased at day 4 after cisplatin-induced AKI; then, this expression was maintained until day 14 and was closely correlated with the histopathological alterations. In contrast, a significantly elevated serum creatinine level was not observed until day 3 after cisplatin-induced AKI. These data were further confirmed in a clinical study. An increased urinary Wnt4 expression was detected earlier than serum creatinine and eGFR in patients with CI-AKI after vascular intervention. This study is the first to demonstrate that increased kidney and urinary Wnt4 expression can be detected earlier than serum creatinine after drug-induced AKI. In particular, urinary Wnt4 could be a potential noninvasive biomarker for monitoring patients with tubular injury.

The cisplatin-induced AKI rat model is a common animal model for researching acute tubular injury. Both necrosis and apoptosis are observed after exposing the tubular cells to cisplatin^36,37^. In the kidney, increased Wnt4 expression is associated with tubulointerstitial fibrosis^38^. However, in our cisplatin-induced AKI rat model, after the Masson staining, we did not observe apparent fibrotic areas (data not shown). In this study, we used immunohistochemistry and immunoblotting to analyze the Wnt4 expression in tubular epithelial cells. At 12 hours after cisplatin administration, Wnt4 was detectable but not prominent in the tubular epithelium; no measurable increase was observed in the serum creatinine level at this time point. Diffuse Wnt4 expression occurred at day 4 and coincided with the peak pathological damage score and serum creatinine level. Simultaneously, a similar trend in kidney Kim-1 expression was observed in the cisplatin-induced AKI model. Expectedly, these data indicated that the upregulation of Wnt4 expression in the injured tubular cells appeared much earlier than the occurrence of significant functional injury in the renal parenchyma. Therefore, Wnt4 may serve as a novel biomarker for monitoring early cisplatin-induced AKI in patients.

At 12 hours after cisplatin administration, although no increase was observed in the serum creatinine level, urinary Wnt4 was detected, and its expression mirrored the changes in renal Wnt4 expression, suggesting that urinary Wnt4 may be secreted from renal tubular epithelial cells. To further investigate the source of Wnt4 in urine, we analyzed the serum by Western blot and found no obvious upregulation of the Wnt4 level in blood (data not shown). However, the molecular mechanisms of Wnt4 secretion into urine remain unknown. Our previous study showed that urinary Wnt4 expression gradually decreased after the injury peak and could still be detected at the repair stage of the IRI model (168 hours after reperfusion). Urinary Wnt4 expression was always parallel to renal Wnt4 expression. However, in our cisplatin-induced AKI model, we found that urinary Wnt4 expression declined faster than tissue Wnt4 expression after the cisplatin-induced injury peak and was absent on day 14 even though a small amount of Wnt4 expression was still detectable in the tubular epithelial cells. Therefore, further studies are necessary to explain the reasons and explore the differences in the patterns of Wnt4 expression among various AKI models. However, the presence of urinary Wnt4 in response to early tubular injury may provide a noninvasive biomarker for AKI patients who are not suitable for renal biopsy.

To verify whether the re-expression of Wnt4 was related to tubular repair and regeneration, we detected Wnt4 co-localization with Ki67, a nuclear antigen that is present during all phases of the cell cycle except G0^39,40^. We found that most Wnt4-positive cells colocalized with Ki67 after cisplatin administration, consistent with previous studies indicating that Wnt4 co-localizes with PCNA, and promoted the cell cycle after ischemia/reperfusion injury^19^. Thus, we speculate that Wnt4 might play a crucial role in tubular repair and regeneration. In addition, some Wnt4-positive cells without Ki67 expression were observed. The underlying mechanisms need to be investigated in a future study. Both proximal and distal tubules undergo cell death during cisplatin nephrotoxicity^41^. In this study, we demonstrated that the Wnt4 expression was enhanced in both injured proximal and distal tubules. Identical results were detected in our previous study using an IRI mouse model^15^. However, Kim-1 is simply expressed in S3 cells of the proximal tubule, whereas NGAL, another precise biomarker of early acute tubular injury, appeared specifically in the distal tubular segments of injured nephrons^42,43^. Therefore, a set of AKI biomarkers (including Wnt4, Kim-1, NGAL *et al*.) could be more helpful for detecting earlier AKI induced by various causes.

Multiple studies have confirmed the renal effects of iodinated intravenous contrast agents. CI-AKI is defined as an acute decrease in renal function after the administration of vascular contrast agents in the absence of other causes^44^. Most CI-AKI patients show a nonoliguric, asymptomatic and transient decrease in renal function that is not easily detected. However, severe renal impairment may lead to oliguria, requiring dialysis and resulting in high mortality^45^. In our clinical study, we found that urinary Wnt4 in CI-AKI patients was detected as early as day 1 after the contrast agent administration, but there was no measurable increase in serum creatinine level at that time point. The finding that urinary Wnt4 appeared earlier than the elevation in serum creatinine level after CI-AKI further validated the clinical utility of urinary Wnt4 as an attractive novel biomarker for the early detection of drug-induced AKI. However, long-term studies are needed to assess the value of urinary Wnt4 in predicting the progression and outcomes of CI-AKI.

Clinical investigations with large sample sizes will be required to fully assess the clinical utility of Wnt4. To explore the utility of Wnt4 in diagnosing acute tubular injury and predicting outcomes, various types of AKI patients should be selected, particularly critically ill patients, whose incidence of AKI varies from 30–70%^46^. In conclusion, our studies demonstrate that Wnt4 is dramatically upregulated after acute, drug-induced tubular injury. During the early phase of AKI, changes in both renal and urinary Wnt4 are detectable before a significant increase in serum creatinine is observed. Moreover, urinary Wnt4 may have a potential to serve as a noninvasive biomarker for identifying early AKI.

## Materials and Methods

### Animals

Male Sprague-Dawley rats (270–300 g) were purchased from the 2^nd^ Affiliated Hospital Laboratories of Harbin Medical University. Rats were housed in an air-conditioned room (22 ± 2 °C; 40–70% relative humidity; 12:12 hour light dark cycle), fed commercial rodent chow, given water ad libitum, and acclimated for 1 week before use. All experimental procedures and animal care protocols were approved by the animal committee of Harbin Medical University. Animal experiments were performed in accordance with the Health Guidelines of the National Institutes for the Care and Use of Laboratory Animals.

### Clinical patients and parameter measurements

All patients recruited for this study were admitted to the Radiology Intervention Department at the 2^nd^ Affiliated Hospital of Harbin Medical University from November 2017 to January 2018. Only patients with normal renal function and urinalysis results were candidates for enrollment in this study. CI-AKI was diagnosed as an increased serum creatinine level of at least 25% above the baseline after the contrast administration and without any other renal injury causes. Basal clinical characteristics, such as demographic data, contrast agent information and some biochemical parameters, were recorded. One day prior to and 1, 3 and 5 days after the contrast administration, blood and first morning urine samples were obtained and centrifuged, and the supernatant was stored at −80 °C until further analysis. The Internal Review Board of Harbin Medical Hospital approved the study protocol, and all patients provided informed consent according to the latest version of the Helsinki Declaration on human research ethics. All methods were performed according to the approved guidelines.

### Cisplatin-induced AKI model

To generate the cisplatin-induced AKI model, cisplatin (Qilu-pharma, Jinan, China) was freshly prepared in 0.9% saline at a concentration of 1 mg/ml, and rats received a single intraperitoneal (i.p.) injection (3 mg/kg) of the solution. The rats in the control group were given an equal volume of normal saline instead of the cisplatin. Rats (n = 6 in each group) were sacrificed under chloral hydrate anesthesia at 12 hours and 1, 2, 3, 4, 5, 6, 14 days after the cisplatin injection. Blood, urine and kidney samples were harvested for further analysis. Urine was collected before euthanasia from each rat, which was housed in a metabolic cage with free access to water but without food. Blood was taken from the abdominal aorta. Urine and blood samples were centrifuged at 3,800 rpm for 15 min, and the supernatant and serum were stored at −80 °C until further analysis. Serum creatinine was measured using an enzymatic method by standard laboratory techniques with an automatic biochemistry analyzer (Cobasc311, Roche, Germany). Renal tissues were prepared for histological study, immunohistochemical examination and molecular biology experiments.

### Tissue collection and preparation

Kidneys from control and treated rats were perfused with ice-cold 0.9% saline at different time points. Then, the right kidney was removed and cut into two halves. One half was fixed in paraformaldehyde/lysine/periodate (PLP) solution for 2 hours, followed by incubation in 18% sucrose overnight^42^. The tissues were then embedded in Tissue-Tek O.C.T. compound and stored at −80 °C. The other half of the right kidney was stored in 10% neutral-buffered formalin for 24 hours, dehydrated in graded ethanol and then embedded in paraffin. The left contralateral kidney, which was used for biochemical analyses, was hemisected and snap-frozen in liquid nitrogen; tissues were stored at −80 °C.

### Histology and immunohistochemistry

Histology and immunohistochemistry were carried out on 2-μm-thick wax sections. Sections on glass slides were first dewaxed and hydrated and then stained with periodic acid-Schiff (PAS) for morphological study. The tubular damage first appeared in the OSOM region but gradually appeared in the cortex; thus, we chose to include only the OSOM region in the statistical analysis. At least ten randomly chosen fields (OSOM region) were evaluated under the microscope (200×, Nikon DS Ri1) for each rat, and an average score was calculated. Tubular injury was defined by epithelial degeneration, brush border loss, cast formation, tubular dilatation and necrosis. Abnormalities were scored by a semiquantitative method: a score of 0 represents an injury area of less than 10% of the field, whereas a score of 1, 2, 3, or 4 represents injury involving 10–25%, 25–50%, 50–75% or >75% of the field, respectively^47^. For Wnt4, Kim-1 and CD68 immunohistochemical staining in tissues, paraffin-embedded kidney sections were deparaffinized, hydrated, and subjected to heat-mediated antigen retrieval, and the endogenous peroxidase activity was ablated by 3% H_2_O_2_. Then, the sections were blocked with 2% BSA in PBS at room temperature for 30 min, followed by incubation with a mouse monoclonal Wnt4 antibody (1:400, Santa Cruz Biotech, Delaware Avenue, CA, USA), a goat polyclonal Kim-1 antibody (1:400, R&D Systems, Minneapolis, MN, USA) or a mouse monoclonal CD68 antibody (1:200, Bio-Rad, Kidlington, UK) overnight at 4 °C. After incubation with horseradish peroxidase-conjugated anti-mouse or anti-goat IgG (ZSGB-BIO, Beijing, China) for 20 min, the sections were developed with a DAB kit (ZSGB-BIO, Beijing, China). The expression levels of kidney Wnt4 and Kim-1 were analyzed using Image-Pro Plus software.

### TUNEL staining

Apoptotic cells were detected by using a terminal deoxynucleotidyl transferase–mediated dUTP nick-end labeling staining kit (Roche, Indianapolis, IN, USA). Images were captured using a Nikon microscope (Tokyo, Japan), processed and analyzed by Image-Pro Plus software.

### Immunofluorescence staining

For immunofluorescence staining, the PLP-fixed and O.C.T.-embedded kidney tissues were sectioned by a cryostat (Thermo Scientific, Cheshire, UK) to 4 μm thick. Then, the cryosections were blocked with 2% BSA in PBS for 30 min and incubated with the following primary antibodies overnight at 4 °C: mouse monoclonal Wnt4 (1:200, Santa Cruz Biotech, Delaware Avenue, CA, USA), goat polyclonal Kim-1 (1:400, R&D Systems, Minneapolis, MN, USA), rabbit monoclonal Ki67 (1:400, Cell Signaling Technology, Danvers, MA, USA, a marker for cell proliferation or regeneration), rabbit polyclonal AQP-1 (1:200, Proteintech, Rosemont, IL, USA, a marker of proximal tubules), goat polyclonal NCCT (1:100, Santa Cruz Biotech, Delaware Avenue, CA, USA, a marker of distal tubules) and rabbit polyclonal NCCT (1:200, Merck, Darmstadt, Germany). Double staining was performed on the same tissue section. On the following day, the sections were washed several times and incubated at room temperature for 1 h with Alexa Fluor 594/488-conjugated secondary antibodies (Jackson ImmunoResearch Laboratories, West Grove, PA) at dilutions of 1:200. Nuclei were stained using 4, 6-diamidino-2-phenylindole (DAPI). Images were captured using a Nikon DS Ri1 camera (Tokyo, Japan) and processed and analyzed by Image-Pro Plus software.

### Western blot analysis

For immunoblotting Wnt4 protein expression in cisplatin-treated rat kidneys and urine samples, the hemisected frozen kidneys were homogenized in radioimmunoprecipitation assay (RIPA) buffer containing 1% Triton X-100, 1% sodium deoxycholate, 0.1% SDS, 1 mM PMSF and proteinase inhibitor cocktail (Roche, Indianapolis, IN, USA) on ice. The supernatants of the kidney lysates or thawed urine samples were collected after centrifugation at 13,500 rpm at 4 °C for 15 min. Protein concentration was determined by bicinchoninic acid protein assay. An equal amount of kidney total protein lysate (40 μg) or centrifuged urine (10 μl) was mixed with SDS-PAGE protein loading buffer, denatured at 80 °C for 10 min and then applied to 12% SDS-polyacrylamide gels for electrophoresis. Separated proteins were transferred to PVDF membranes by standard techniques. The blots were blocked in 5% nonfat dry milk in TBST for 60 min and then incubated with primary antibodies against Wnt4 (1:400, Santa Cruz Biotech, Delaware Avenue, CA, USA) or β-actin (1:1000, Santa Cruz Biotech, Delaware Avenue, CA, USA) overnight at 4 °C, followed by an incubation with secondary antibody goat anti-mouse IgG (1:8000, Jackson ImmunoResearch Laboratories, WestGrove, PA, USA) at room temperature for 60 min. Finally, the proteins were visualized using Super ECL Reagent (HaiGene, Harbin, China), examined using a luminescence image analyzer (GE Healthcare Bio-Sciences AB, Uppsala, Sweden) and quantified by ImageJ software. Full-sized blots are shown in Fig. S4.

### Enzyme-linked immunosorbent assay (ELISA)

Urinary Wnt4 and Kim-1 from cisplatin-treated rats and CI-AKI patients was measured by ELISA using commercially available test kits (CUSABIO, Wuhan, China) according to the manufacturer’s protocol and was normalized to urinary creatinine (uCr).

### Statistical analyses

All results are presented as the means ± SD or the medians (interquartile ranges). Statistical differences were analyzed using one-way analysis of variance (ANOVA), nonparametric tests, or the Chi-square test, and *p* values of <0.05 or 0.01 were considered significant. Pearson or Spearman correlation analysis was used for comparisons between two variables.

### Data availability

The datasets generated during and/or analyzed during the current study are available from the corresponding author on reasonable request.

## Electronic supplementary material

Supplementary figures and figure legends
