# Supplementary material for: Wnt4 is significantly upregulated during the early phases of cisplatin-induced acute kidney injury
Source: Sci Rep. 2018 Jul 12;8:10555. doi: 10.1038/s41598-018-28595-4 (PMC6043520; doi:10.1038/s41598-018-28595-4)
Supplement: Supplementary file 1 — Supplementary figures and figure legends [file 41598_2018_28595_MOESM1_ESM.docx]

**Wnt4 is significantly upregulated during the early phases of cisplatin-induced acute kidney injury**

YI-XIN HE M.D., TIAN-TIAN DIAO M.D., SHU-MIN SONG M.D., PhD, CEN-CEN WANG M.D., PhD, YU WANG M.D., CHUN-LAN ZHOU M.D., YI-BING BAI M.D., SHAN-SHEN YU M.D., XUAN MI M.D., XIN-YU YANG M.D., QIU-JU WEI M.D., BING LI^*^ M.D., PhD.

*Department of Nephrology, the Second Affiliated Hospital of Harbin Medical University, Harbin, People’s Republic of China*

^*^**Correspondence**: Prof. Bing Li, Department of Nephrology, 2^nd^ Affiliated Hospital of Harbin Medical University, 246 Xuefu Road, Nangang District, Harbin 150086, P.R.C. Telephone: 0086-451-86297145. E-mail: [icecreamlee@hotmail.com](mailto:icecreamlee@hotmail.com)

**Supplementary Figures and Figure Legends**

**
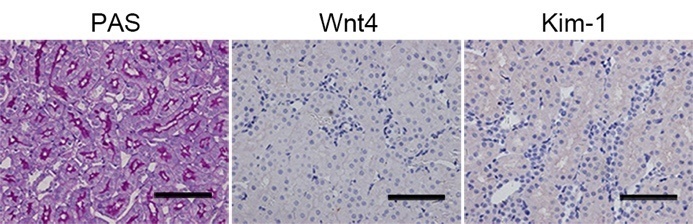
**

**Supplementary Figure S1.** Periodic acid–Schiff staining and immunostaining of Wnt4 and Kim-1 in the kidneys of healthy adult rats.


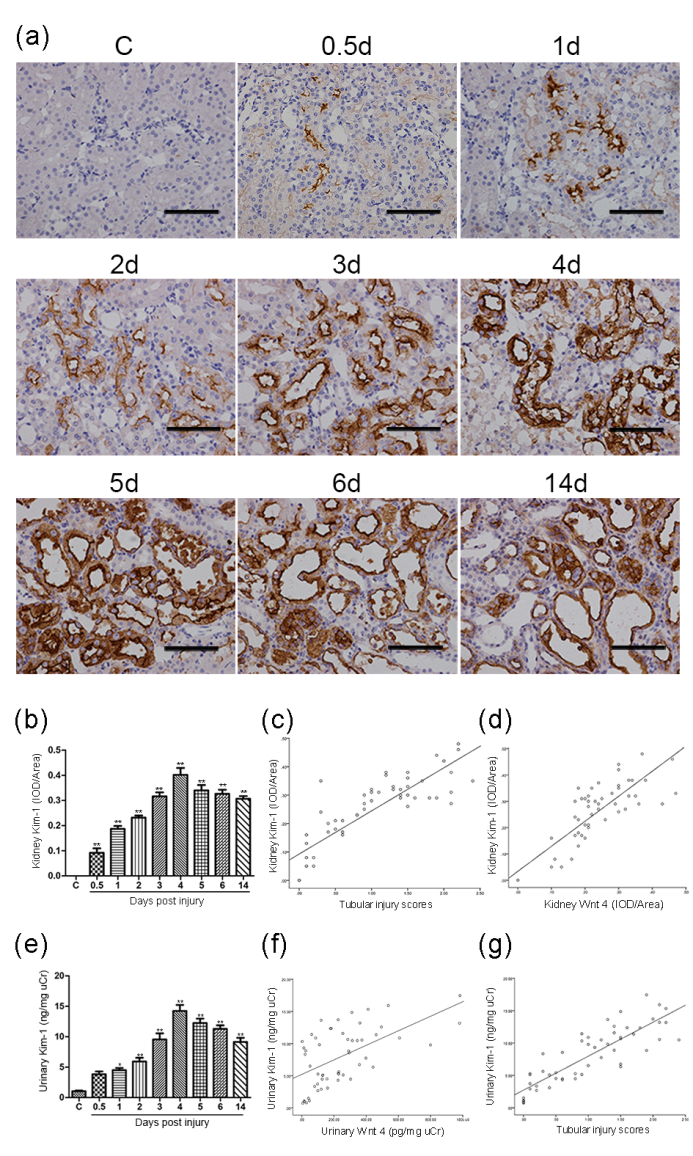


**Supplementary Figure S2.** Renal and urinary Kim-1 expression is significantly upregulated during the early stage of cisplatin-induced AKI and is correlated with tubular injury and Wnt4 expression. (a) Representative immunohistochemical images showing kidney Kim-1 expression (brown) in the control and cisplatin-induced AKI rats at different time points (magnification, 200x). Bar=100μm. (b) Quantification of kidney Kim-1 expression in each group. Data are presented as the IOD/ Kim-1-positive areas, as analyzed using Image-Pro Plus software. (c) Correlation between kidney Kim-1 expression and tubular injury by immunohistochemical staining (r^2^=0.849, *p*<0.01). (d) Correlation between kidney Kim-1 expression and kidney Wnt4 expression by immunohistochemical staining (r^2^=0.845, *p*<0.01). (e) ELISA analysis of urinary Kim-1 normalized to uCr in each group. (f) Correlation between urinary Kim-1 and urinary Wnt4 (r^2^=0.573, *p*<0.01). (g) Correlation between urinary Kim-1 and tubular injury (r^2^=0.858, *p*<0.01). **p*<0.05, ***p*<0.01 versus the control group (n=6). uCr, urinary creatinine.


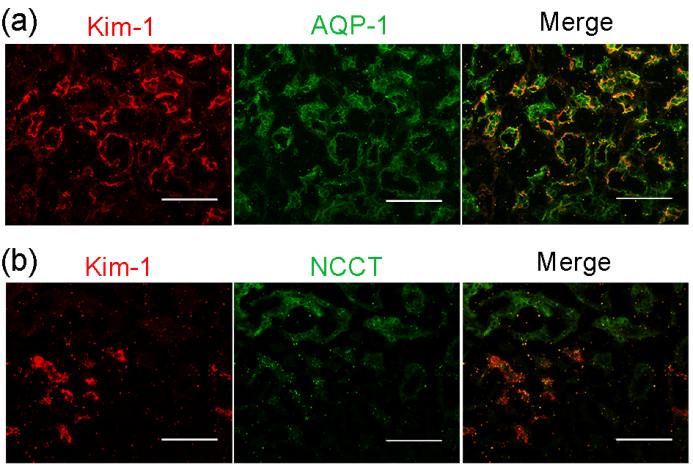


**Supplementary Figure S3.** Kim-1 is expressed in injured proximal tubules after cisplatin treatment. (a) Co-staining of Kim-1 and the proximal tubular marker AQP-1. (b) Co-staining of Kim-1 and the distal tubular marker NCCT.


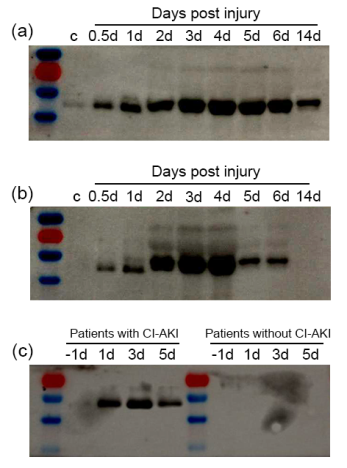


**Supplementary Figure S4.** Full-sized blots are shown. (a) Kidney Wnt4 expression in cisplatin-induced AKI rats. (b) Urinary Wnt4 expression in cisplatin-induced AKI rats. (c) Urinary Wnt4 expression in patients with CI-AKI and patients without CI-AKI.
